# Profiling and functional analysis of differentially expressed circular RNAs identified in foot-and-mouth disease virus infected PK-15 cells

**DOI:** 10.1186/s13567-022-01037-w

**Published:** 2022-03-21

**Authors:** JinKe Yang, Bo Yang, Yong Wang, Ting Zhang, Yu Hao, HuiMei Cui, DengShuai Zhao, XingGuo Yuan, XueHui Chen, ChaoChao Shen, WenQian Yan, HaiXue Zheng, KeShan Zhang, Xiangtao Liu

**Affiliations:** State Key Laboratory of Veterinary Etiological Biology, National Foot-and-Mouth Disease Reference Laboratory, Lanzhou Veterinary Research Institute, Chinese Academy of Agricultural Sciences, Lanzhou, 730046 China

**Keywords:** FMDV, RNA-Seq, PK-15 cells, circRNA, differential expression, analysis

## Abstract

Circular RNAs (circRNAs) are a new type of endogenous noncoding RNA that exhibit a variety of biological functions. However, it is not clear whether they are involved in foot-and-mouth disease virus (FMDV) infection and host response. In this study, we established circRNA expression profiles in FMDV-infected PK-15 cells using RNA-seq (RNA-sequencing) technology analysis. The biological function of the differentially expressed circRNAs was determined by protein interaction network, Gene Ontology (GO), and Kyoto Encyclopedia of Gene and Genome (KEGG) pathway enrichment. We found 1100 differentially expressed circRNAs (675 downregulated and 425 upregulated) which were involved in various biological processes such as protein ubiquitination modification, cell cycle regulation, RNA transport, and autophagy. We also found that circRNAs identified after FMDV infection may be involved in the host cell immune response. RNA-Seq results were validated by circRNAs qRT-PCR. In this study, we analyzed for the first time circRNAs expression profile and the biological function of these genes after FMDV infection of host cells. The results provide new insights into the interactions between FMDV and host cells.

## Introduction

Circular RNAs (circRNAs) belong to a class of non‐coding RNAs that are widespread in the cytoplasm of eukaryotic cells and are structurally and functionally different from linear RNA molecules [1]. They are covalently closed-loop RNA molecules that are formed by back-splicing of the 5′ and 3′ ends of the primary transcript. They have a strong structural stability, tissue, and spatiotemporal specificity [2, 3]. To date, three types of circRNA molecules have been reported including circRNAs generated by reverse exon splicing, circRNAs that form by intronic lasso, and circRNAs consisting of both introns and exons [4–7]. Previously, circRNAs were considered by-products of abnormal splicing during transcription; however, with the rapid development of high-throughput sequencing technologies and bioinformatics, there is growing evidence that circRNAs are involved in regulating a variety of important physiological functions [8]. Most circRNAs are composed of exons and are located in the cytoplasm, indicating that they function as protein regulators in the translation and modification of proteins [7, 9–12]. Recently, it has been shown that circRNAs act as sponges for microRNAs (miRNAs), which act as competing endogenous RNAs (ceRNAs) to regulate post-transcriptional gene expression events [13–15]. Other studies have found that circRNAs play important regulatory roles in pathological processes such as neurological diseases [16] and cancer [17, 18]. In addition, because of the low molecular weight of circRNAs, they are transported by extracellular vesicles, such as nanoparticles and exosomes. They are now widely studied and considered as molecular markers, therapeutic targets, and drug carriers in a variety of diseases [19].

As novel regulatory molecules, circRNAs mediate the regulation of viral infections and the cellular immune response, which provide a new perspective for understanding virus–cell interactions. Recently, several studies have shown that circRNAs are also involved in the regulation of virus–host cell interactions as well as in the antiviral cell immune response [5, 20]. For example, during H1N1 influenza A virus (IAV) infection, circGATAD2A overexpression in the host promotes replication of the H1N1 IAV virus [21]. Hepatitis C virus infection induces host circRNAs to exert nonsense-mediated decay and inhibit viral replication [22]. Viruses can also use circRNAs to interfere with the host antiviral immune response and help to escape immune surveillance and antiviral immunity. Interestingly, when viral invasion occurs, some circRNAs inhibit immune cell activation; however, when circRNAs are degraded by RNase L, they subsequently regulate autoimmune disease or viral infection clearance by activating PKR activation and downstream cascade responses [23]. Although studies have demonstrated that circRNAs are involved in the regulation of host cells after viral infection, there is a lot to unravel to understand the role that circRNAs play in virus–host interactions and viral pathogenesis.

Foot-and-mouth disease (FMD) is a highly contagious viral disease that occurs in livestock worldwide. FMD is caused by the foot-and-mouth disease virus (FMDV), which mainly infects cloven-hooved animals [24]. Pathological blisters appear in the oral mucosa, extremities, and breasts as the main clinical symptoms, and animals may die from severe infections. This causes significant losses to the animal husbandry industry and the economy [25]. FMDV has a genome of approximately 8.5 kb and is a single-stranded positive-sense RNA virus of the genus *Aphthovirus*, within the family *Picornaviridae*. It contains an open reading frame (ORF) that encodes four structural proteins (VP1, VP2, VP3, and VP4) and 10 nonstructural proteins (L^pro^, 2A, 2B, 2C, 3A, 3B1, 3B2, 3B3, 3C, and 3D) [26–28]. FMDV is divided into seven serotypes (A, O, C, SAT 1, SAT 2, SAT 3, and Asia 1) according to geographical distribution and each serotype has a wide range of antigenic characteristics which do not elicit effective cross-protection. The high incidence and extensiveness worldwide make the prevention and control of FMD a great challenge [29]. Several studies have shown that FMDV is able to escape the host innate immune response through multiple pathways. For example, FMDV antagonizes host antiviral interferon production by inducing PERK and AKT-MTOR signaling to regulate autophagy [30, 31]. FMDV L^pro^ is able to target ISG15 by specifically cleaving the peptide bonds before the C-terminal Gly-Gly motif. This disrupts the ubiquitination modification process and prevents it from recognizing viral proteins, thereby preventing the initiation of innate immune response signals [32]. FMDV also negatively regulates the activation of the INF-β signaling pathway by inhibiting the phosphorylation of IRF3 and nuclear translocation through the VP1 protein [33].

Host circRNAs expression and potential role during FMDV infection are unknown. Pigs are the primary natural reservoir of FMDV and have been used as a challenge model [34]. In this study, we used RNA-Seq technology with Illumina HiSeq platform to analyze the characteristics of differentially expressed circRNAs and the biological function of parental genes in PK-15 cells after FMDV infection. RNA- reliability Seq results was verified by qRT-PCR. The results provide new clues for understanding the interaction between FMDV and host cells from the perspective of circRNAs.

## Materials and methods

### Cell culture and virus infection

Porcine kidney cells (PK-15 cells) were cultured at 37 °C in a 5% CO_2_ humidified atmosphere in Dulbecco’s modified Eagle’s medium (DMEM, Gibco, USA) supplemented with 10% heat-inactivated fetal bovine serum (FBS, Gibco, USA) and 1% antibiotic/antimycotic solution (100U/mL penicillin, 100 μg/mL streptomycin). FMDV serotype A (FMDV A/GD/MM) was provided and stored by the OIE/National Foot and Mouth Disease Reference Laboratory (Lanzhou, Gansu, China). To analyze cell response to FMDV infection, 80% confluent PK-15 cells were inoculated with FMDV or mock infected. After 8 h of infection, both the cell supernatant and precipitate were used for further analysis. All the virus-related experiments were conducted in the Biosafety Level-3 (BSL-3) laboratory of Lanzhou Veterinary Research Institute according to the standard protocols and biosafety regulations provided by the Institutional Biosafety Committee.

### RNA extraction, library construction, and RNA-sequencing

Total RNA was extracted from both FMDV-infected and mock-infected cells using TRIzol reagent (Invitrogen, USA). Total RNA was quantified and the quality assessed using an Agilent 2100 Bioanalyzer (Agilent Technologies, USA), and 1 μg total RNA with a RIN value above 8 was used for library preparation. Pair-End index libraries were constructed according to the manufacturer’s protocol (NEBNext® Ultra™RNA Library Prep Kit for Illumina®). The libraries with human circRNAs sequencing was multiplexed and loaded onto an Illumina HiSeq instrument (Illumina, USA) according to the manufacturer’s instructions. RNA-seq was performed using an Illumina HiSeq to get the raw data (raw reads). Sequencing was carried out in a 2 × 150 paired-end (PE) configuration. All sequences were processed and analyzed by the Shanghai Yuanxin Biomedical Technology Company.

### circRNA sequence prediction

After the libraries were sequenced, the PE reads for each sample were mapped to a reference genome (GRCh38, Ensemble 91) using TopHat2 [35, 36].

### Differential expression analysis of circRNAs

DE circRNAs were identified and analyzed using DESeq software based on a negative binomial distribution [37, 38]. Differentially expressed (DE) genes were detected with |log_2_ (fold change) |≥ 1 and *P* value ≤ 0.05.

### Gene Ontology and Kyoto Encyclopedia of Gene and Genome pathway analysis

Gene Ontology (GO) analysis was used to annotate the biological processes involved and the functions associated with the parental genes of the circRNAs (molecular functions, cellular components, and biological processes) [39–41]. Kyoto Encyclopedia of Gene and Genome (KEGG) enrichment using hypergeometric test was also conducted to predict the involvement of cellular pathways targeted by circRNAs during FMDV infection [42]. The GO and KEGG pathways identified with corrected *P* values ≤ 0.05 were considered significantly enriched.

### Protein interaction map of parental genes of differentially expressed circRNAs

Protein function related network analysis of circRNAs genes with significant differential expression was done using the Retrieval of Interacting Genes/Proteins (STRING v10) database and web tool [43]. The DE genes were screened with |log_2_ (fold change) |≥ 2 and* P* value ≤ 0.05.

### Differentially expressed circRNAs preparation and qRT-PCR analysis

To validate the accuracy of the sequencing results and screen the partially differentially expressed circRNAs, we designed divergence primers based on the predicted sequences of the circRNAs phenology. The reliability of the sequencing results was verified by RT-qPCR based on selected circRNAs. To remove interference from linear RNA, FMDV infected group samples were treated with Ribonuclease R (RNase R), and the reaction mixture was incubated at 37 °C for 30 min and inactivated in a water bath at 72 °C for 10 min. For the control group, RNA was not treated with RNase R. After that, cDNA was synthesized using reverse transcription kit (Takara, Dalian, China) following the manufacturer’s instructions. The expression of differential circRNAs was verified using the qRT-PCR assay. qRT-PCR was performed at 20 µL reaction volume, including 10 µL SYBR Green Master Mix, 1 µL PCR primers (forward and reverse, respectively), 5 µL nuclease-free water, and 3 µL cDNA. The reaction was performed at 95 °C for 2 min, followed by 45 cycles with 95 °C for 10 s and 60 °C for 10 min. The GAPDH of pigs was used as a reference house-keeping gene. All reactions were run in triplicate. The 2^−ΔΔCt^ method was used to measure expression level of target circRNAs.

### Statistical analysis

Statistical significance analysis was performed by the Student’s *t*-test and *P* values ≤ 0.05 were considered statistically significant.

## Results

### Identification and classification of circRNAs

**Figure 1:**
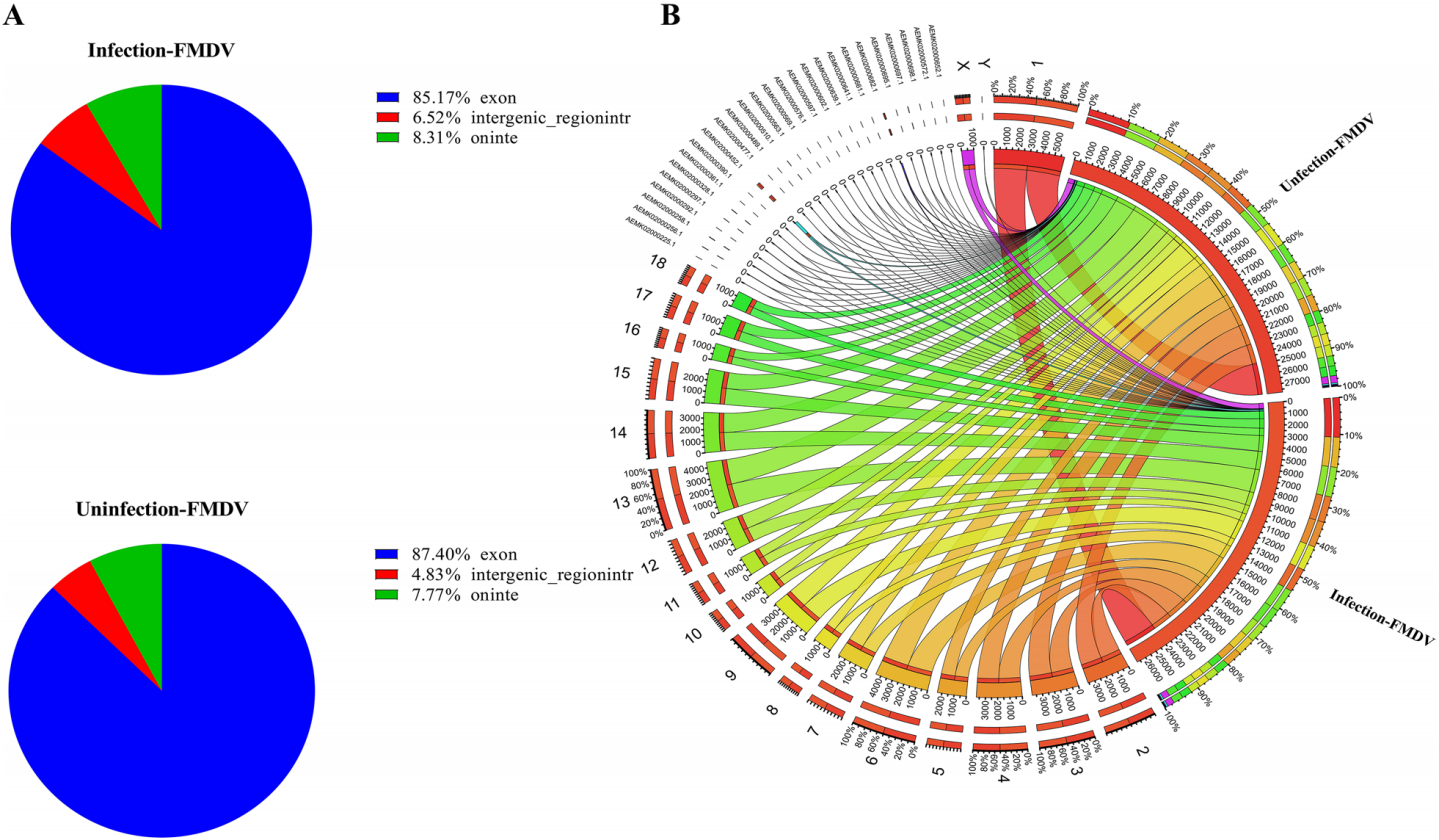
**circRNA sequencing data.** (**A**) Categories of circRNAs in PK-15 cells infected with FMDV and mock groups. (**B**) Circos plots: the outline is the chromosome coverage of all circRNAs infected with FMDV along with the mock groups, whereas the inside represents the proportion of circRNAs in the reference genome.

To identify differentially expressed circRNAs in FMDV-infected PK-15 cells, FMDV-infected cells and mock-infected cells were sequenced separately using the Illumina HiSeq platform. As shown in Table 1, a total of 147084824 and 140485672 circRNA raw sequence numbers were identified in FMDV-infected and mock-infected groups, respectively. After filtering and screening, 146011792 and 139140012 high quality sequences were obtained for analysis. Candidate circRNA sequences were mapped to the corresponding genomes for comparison and the FMDV-infected and mock-infected group rates were 87.18% (133109434/152684665) and 91.11% (131008908/143792709), respectively. The Q30 values for the samples were 94.83% and 95.16%. In addition, the circRNA types were identified and characterized by CIRI2 sequence alignment. Figure 1A shows that the circRNAs in the FMDV-infected and mock-infected groups were mainly derived from exons (85.17%, 87.40%), introns (8.31%, 7.77%), and intergenic regions (6.52%, 4.83%). Circos plots showed the distribution of circRNAs on chromosomes from the FMDV-infected and mock-infected groups (Figure 1B).

**Table 1:** **Summary of Illumina Hiseq sequencing data for circRNAs after infection with FMDV along with the mock group**

Categories	FMDV infection	NC
Raw reads (items)	147084824	140485672
Clean reads (items)	146011792	139140012
GC content (%)	48.71	53.26
Q30 (%)	94.83	95.16
Mapped rate (%)	87.18 (133109434/152684665)	91.11 (131008908/143792709)

### Screening of differentially expressed circRNAs

**Figure 2:**
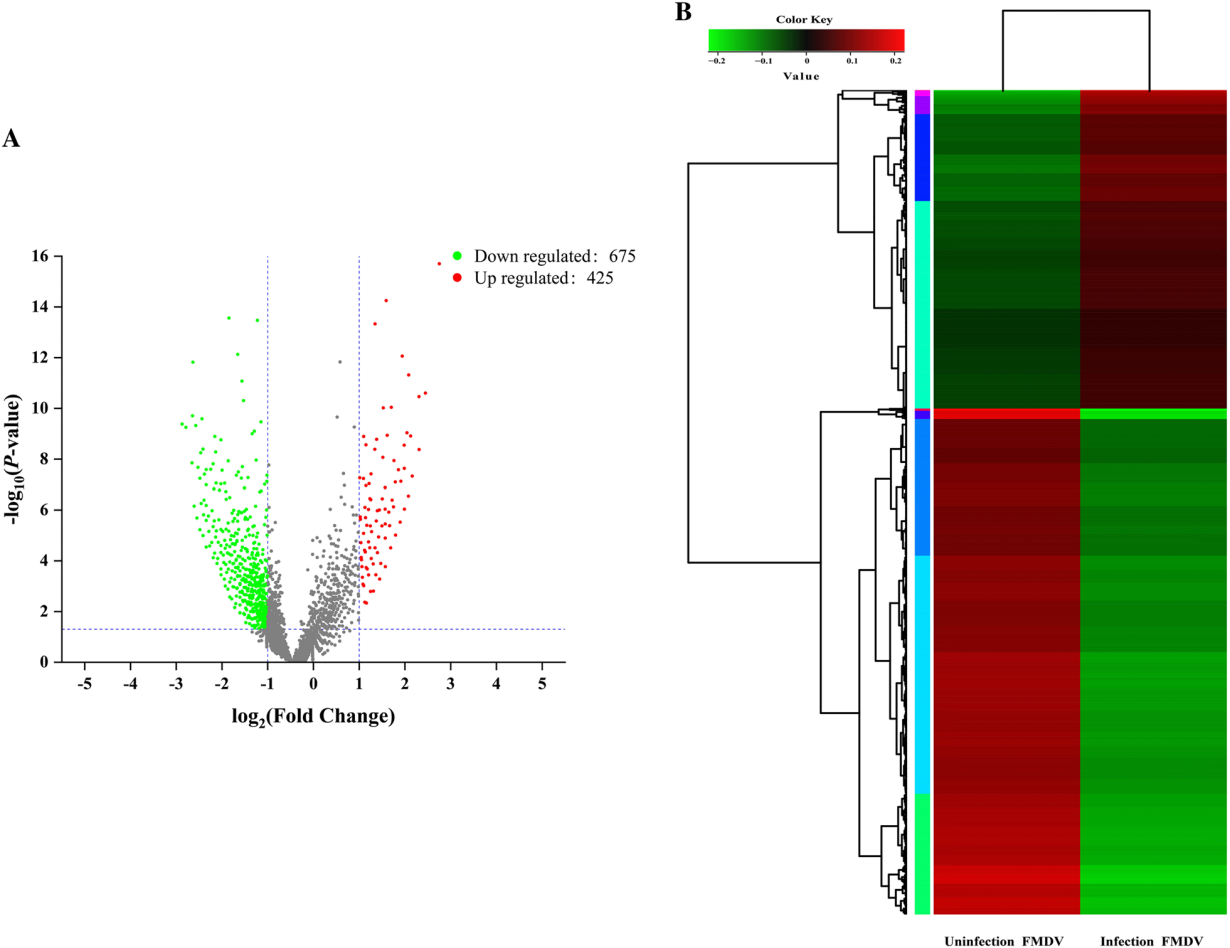
**Volcano plot and heatmap analysis of differentially expressed circRNAs after FMDV infection.** (**A**) The red, green, and gray dots represent upregulated, downregulated, and not significantly regulated circRNAs respectively. The *x*-axis indicates the fold-changes of differentially expressed circRNAs; the greater the absolute *x*-value, the greater the fold change. The *y*-axis represents the significance of the differentially expressed circRNAs; the greater the *y*-value, the smaller the *p* value. (**B**) Red indicates higher expression and green indicates lower expression. Each row represents a circRNA parental gene, each column represents a sample, and the tree diagram of the gene cluster is to the left.

With the development of sequencing technologies, high-throughput circRNA sequencing is now a standard method to evaluate circRNAs expression and it is widely used to deduce the effects of host genes in various diseases [44]. There were 1100 differentially expressed circRNAs obtained from PK-15 cells after FMDV infection, of which 425 circRNAs were upregulated and 675 were downregulated. Because of the specificity of circRNAs, SRPBM (Spliced Reads per Billion Mapping) Trans-splicing reads are usually used to estimate circRNA expression. We illustrated the whole expression trend and distribution of all differentially expressed circRNAs using volcano plots (Figure 2A) and clustering heatmaps (Figure 2B). The results showed that FMDV infection of PK-15 cells altered the intracellular circRNAs expression profile, resulting in deregulated expression of multiple circRNAs. This suggests that circRNAs may have a biological function during the cellular response to virus infection.

### Functional analysis of differentially expressed circRNAs

**Figure 3:**
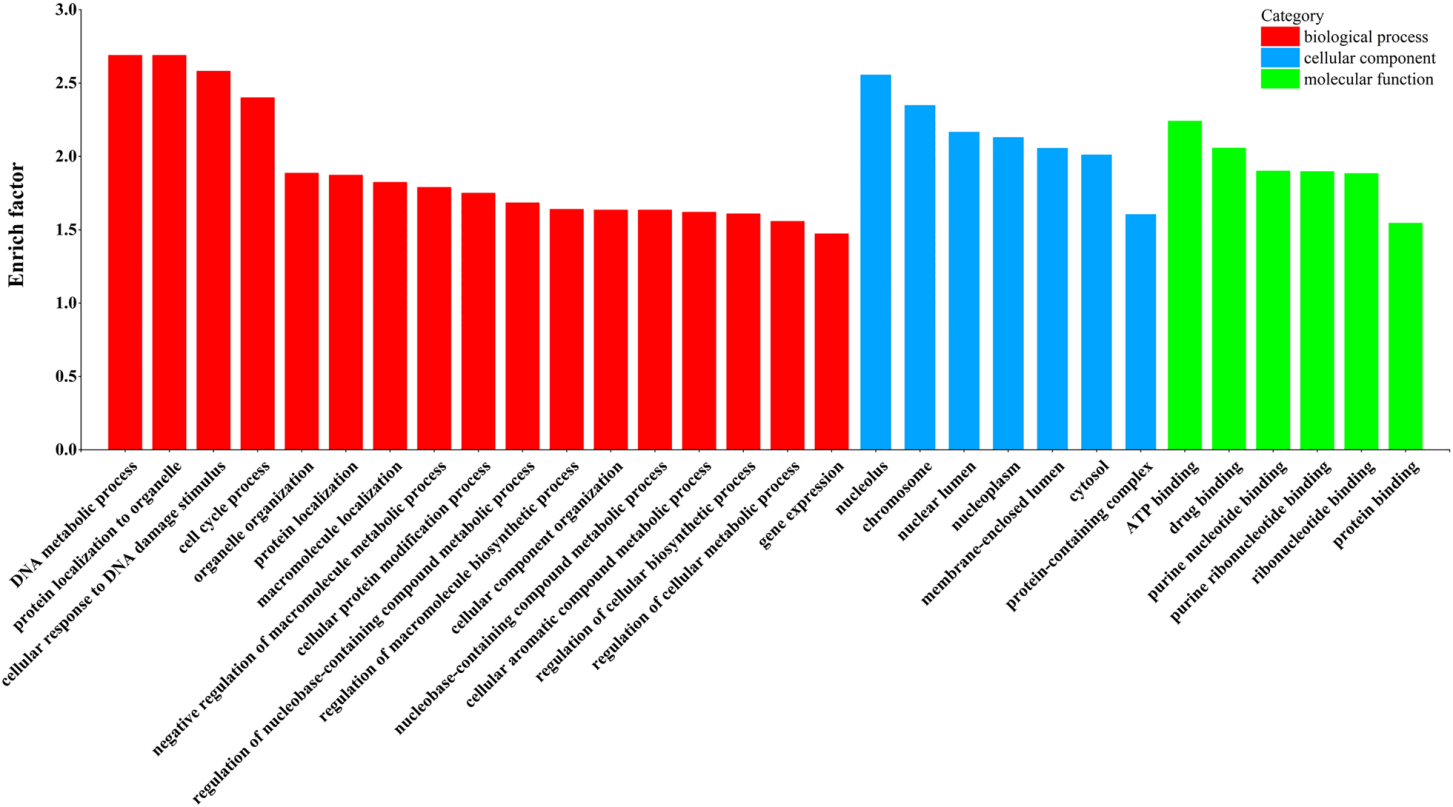
**Top 30 Gene Ontology (GO) functional classifications and enrichment analysis of differentially expressed circRNAs genes.** The *x*-axis indicates the functional description. The *y*-axis indicates the enrichment significance. GO functional classification from biological process (BP), cellular component (CC), and molecular function (MF).

**Figure 4:**
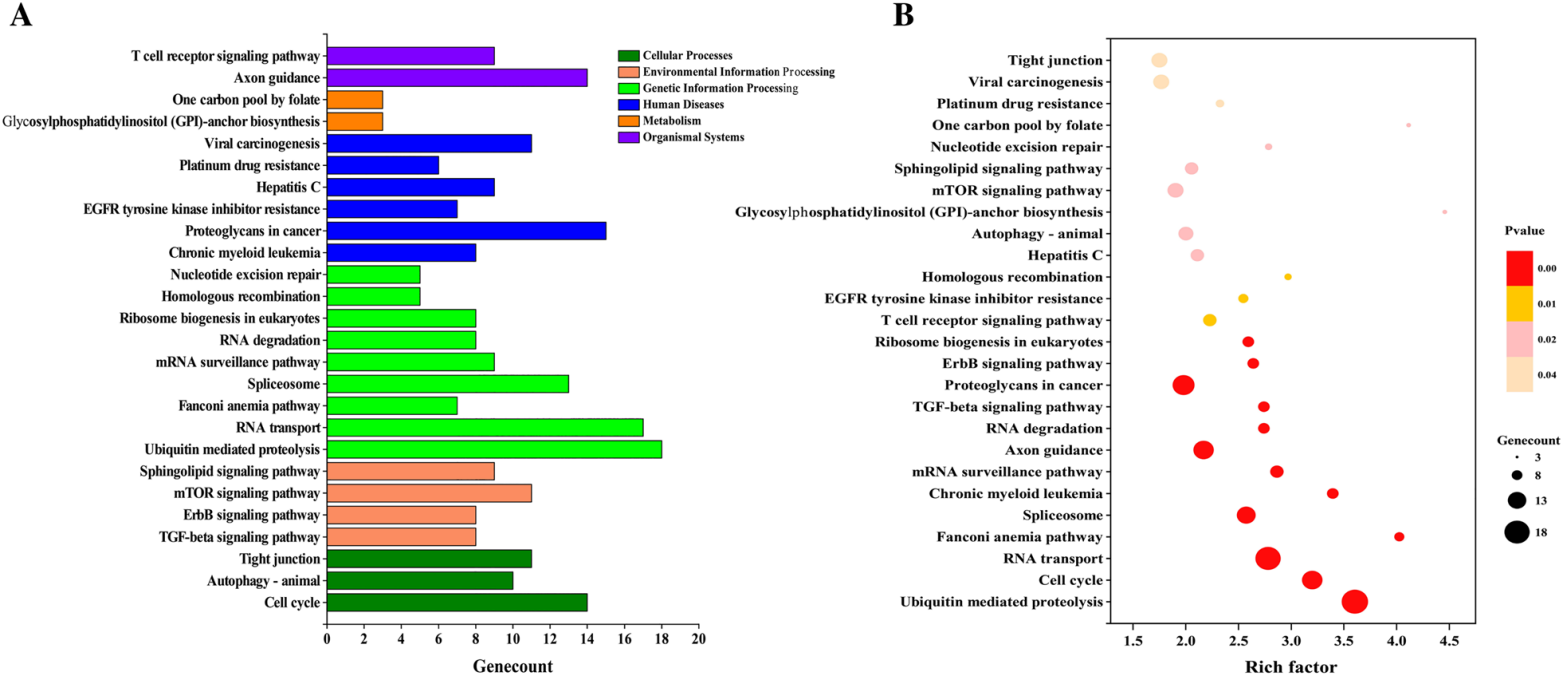
**KEGG classification and pathway enrichment analysis of differentially expressed circRNA genes.** (**A**) KEGG classifications of differentially expressed circRNA genes. The *x*-axis indicates the number of differentially expressed circRNA genes and the *y*-axis represents KEGG terms. (**B**) Top 30 pathway enrichment results for differentially expressed circRNA genes. The *x*-axis indicates the degree of enrichment, whereas the *y*-axis represents the functional descriptions of the enriched pathways.

To further reveal the potential biological functions of FMDV-induced differentially expressed circRNAs, we performed GO and KEGG pathway functional enrichment analysis for selected differentially expressed circRNA genes. GO is a widely used bioinformatics database that includes genes and gene product information for all species [45]. First, GO analysis was performed on differentially expressed circRNAs parental genes. The results showed that differentially expressed circRNAs were mainly involved in nucleic acid metabolism, cellular metabolism, and protein synthesis and modification (Figure 3). It has been demonstrated that circRNAs can actively participate in regulating cellular metabolic processes [46, 47]. Because different genes cooperate with one another to regulate biological processes, further understanding of the potential biological mechanism of differentially expressed circRNAs may be gleaned through KEGG pathway enrichment analysis. From the figures, it is clear that most of the differentially expressed circRNAs are closely associated with multiple signaling pathways including protein ubiquitination, degradation, cell cycle regulation, RNA transport, autophagy signaling, mTOR signaling, T cell receptor signaling, and nucleotide excision repair (Figure 4). Overall, we found that differentially expressed circRNA genes may regulate multiple pathways, such as nucleic acid metabolism, cellular metabolism, signal transduction, and the immune response to regulate the cellular response to viral infection.

**Figure 5:**
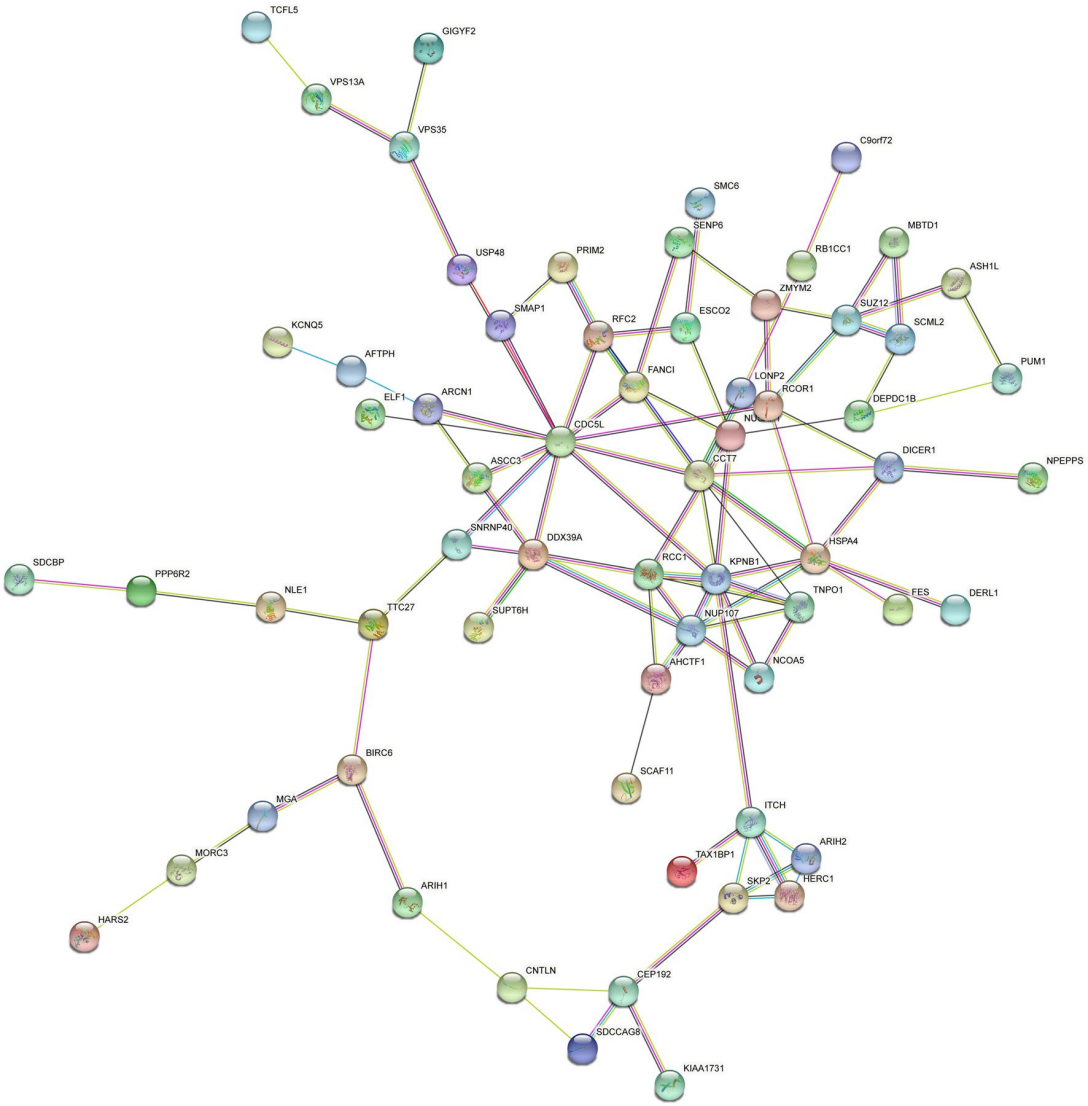
**Protein function interaction network diagram of differentially expressed circRNA genes.** These genes are involved in various biological processes including nucleic acid metabolism, protein ubiquitination modifications, and cell cycle regulation.

Based on gene function analysis using GO and KEGG (Tables 2 and 3), we explored the regulatory functions of these differentially expressed circRNA genes at the translation level. We screened 64 genes with significantly differentially expressed circRNAs, 8 were significantly upregulated and 56 were downregulated (*p* value < 0.05 and |log_2_(FC)|> 2) using the STRING for protein interaction analysis (Figure 5). Interestingly, the results showed that these genes were primarily involved in regulating biological processes such as the protein ubiquitination, cell cycle, and nucleic acid metabolism. In vivo, ubiquitination is a key signaling and defense mechanism for host cells to detect and respond to viral infection, inducing the initiation of immune response signaling pathways against viral invasion. Based on the protein interaction network, circRNAs may play a role in the host immune response induced by FMDV infection.

**Table 2:** **Differentially expressed upregulated circRNAs (*p*** **value < 0.05)**

Circ RNA ID	Chromosome	Log_2_FC	Start	End	Type	Original gene	Strand
1:205515399–205516988	1	2.31	205515399	205516988	Exon	CNTLN	−
9:45886507–45886672	9	2.31	45886507	45886672	Exon	ARCN1	+
11:25838073–25838397	11	2.16	25838073	25838397	Exon	ELF1	+
4:94369484–94406163	4	2.08	94369484	94406163	Exon	ASH1L	+
1:230276437–230292278	1	2.08	230276437	230292278	Exon	VPS13A	+
12:23921820–23923094	12	2.08	23921820	23923094	Exon	KPNB1	+
1:90220788–90238411	1	2.04	90220788	90238411	Exon	SENP6	−
6:37967643–37975235	6	2.04	37967643	37975235	Exon	VPS35	+

**Table 3:** **Differentially expressed downregulated circRNAs (*p*** **value < 0.05)**

Circ RNA ID	Chromosome	Log_2_FC	Start	End	Type	Original gene	Strand
3:77371816–77378404	3	− 2.02	77371816	77378404	Exon	AFTPH	−
10:15051106–15056946	10	− 2.19	15051106	15056946	Exon	AHCTF1	+
7:60719877–60732585	7	− 2.65	60,719,877	60,732,585	Exon	ARIH1	−
13:31573092–31,575948	13	− 2.46	31573092	31575948	Exon	ARIH2	+
1:67439729–67445988	1	− 2.39	67439729	67445988	Exon	ASCC3	−
3:107127683–107131029	3	− 2.79	107127683	107131029	Exon	BIRC6	−
10:38395600–38396087	10	− 2.27	38395600	38396087	Exon	C9orf72	+
3:69536711–69540489	3	− 2.61	69536711	69540489	Exon	CCT7	−
7:39403319–39407636	7	− 2.27	39403319	39407636	Exon	CDC5L	+
6:96667496–96668320	6	− 2.19	96667496	96668320	Exon	CEP192	−
9:26111481–26112646	9	− 2.34	26111481	26112646	Exon	CEP295	+
2:64840343–64840811	2	− 2.22	64840343	64840811	Exon	DDX39A	+
16:39505968–39516507	16	− 2.24	39505968	39516507	Exon	DEPDC1B	−
4:16323825–16335370	4	− 2.08	16323825	16335370	Exon	DERL1	+
7:116401757–116406130	7	− 2.11	116401757	116406130	Exon	DICER1	−
14:11503553–11515969	14	− 2.27	11503553	11515969	Exon	ESCO2	+
7:54870349–54875452	7	− 2.19	54870349	54875452	Exon	FANCI	+
7:53476795–53477217	7	− 2.19	53476795	53477217	Exon	FES	+
15:133280528–133283709	15	− 2.46	133280528	133283709	Exon	GIGYF2	+
2:142406868–142408143	2	− 2.16	142406868	142408143	Intron	HARS2	+
1:108248956–108249911	1	− 2.19	108248956	108249911	Exon	HERC1	+
2:135356735–135368467	2	− 2.28	135356735	135368467	Exon	HSPA4	+
17:37838591–37854328	17	− 2.19	37838591	37854328	Exon	ITCH	+
1:52764469–52800752	1	− 2.02	52764469	52800752	Exon	KCNQ5	+
6:36387420–36416154	6	− 2.41	36387420	36416154	Exon	LONP2	−
12:27490741–27499620	12	− 2.40	27490741	27499620	Exon	MBTD1	−
1:129601956–129602162	1	− 2.64	129601956	129602162	Exon	MGA	−
13:200017893–200023406	13	− 2.14	200017893	200023406	Exon	MORC3	+
17:48239458–48241671	17	− 2.61	48239458	48241671	Exon	NCOA5	−
12:40037309–40038184	12	− 2.16	40037309	40038184	Exon	NLE1	+
12:23859739–23869452	12	− 2.34	23859739	23869452	Exon	NPEPPS	+
5:32992793–33000625	5	− 2.52	32992793	33000625	Exon	NUP107	+
1:130012096–130015941	1	− 2.34	130012096	130015941	Exon	NUSAP1	−
5:313664–341527	5	− 2.27	313664	341527	Exon	PPP6R2	−
7:28373900–28381978	7	− 2.19	28373900	28381978	Exon	PRIM2	−
6:87615275–87630082	6	− 2.63	87615275	87630082	Exon	PUM1	−
4:77612564–77618720	4	− 2.07	77612564	77618720	Exon	RB1CC1	+
6:85556400–85558524	6	− 2.02	85556400	85558524	Exon	RCC1	+
11:713800–716256	11	− 2.02	713800	716256	Exon	ZMYM2	+
3:11377601–11380582	3	− 2.34	11377601	11380582	Exon	RFC2	−
5:76844512–76857881	5	− 2.02	76844512	76857881	Exon	SCAF11	−
X:14780708–14801436	X	− 2.21	14780708	14801436	Exon	SCML2	−
4:74305210–74307728	4	− 2.55	74305210	74307728	Exon	SDCBP	−
10:16216508–16239910	10	− 2.02	16216508	16239910	Exon	SDCCAG8	+
16:21482127–21485938	16	− 2.30	21482127	21485938	Exon	SKP2	+
1:50832070–50838859	1	− 2.02	50832070	50838859	Exon	SMAP1	+
3:120120935–120125630	3	− 2.16	120120935	120125630	Exon	SMC6	+
6:87847020–87874230	6	− 2.41	87847020	87874230	Exon	SNRNP40	−
12:44919705–44920437	12	− 2.14	44919705	44920437	Exon	SUPT6H	+
12:42993597–42998085	12	− 2.19	42993597	42998085	Exon	SUZ12	−
18:44873297–44897643	18	− 2.41	44873297	44897643	Exon	TAX1BP1	−
17:62094151–62114034	17	− 2.19	62094151	62114034	Exon	TCFL5	−
16:49107259–49112899	16	− 2.17	49107259	49112899	Exon	TNPO1	+
3:106854519–106888705	3	− 2.19	106854519	106888705	Exon	TTC27	−
6:79779444–79787224	6	− 2.21	79779444	79787224	Exon	USP48	−
AEMK02000452.1:1423806–1456508	AEMK02000452.1	− 2.09	1423806	1456508	Exon	RCOR1	+

### Validation of the differentially expressed circRNAs by qRT-PCR

**Figure 6:**
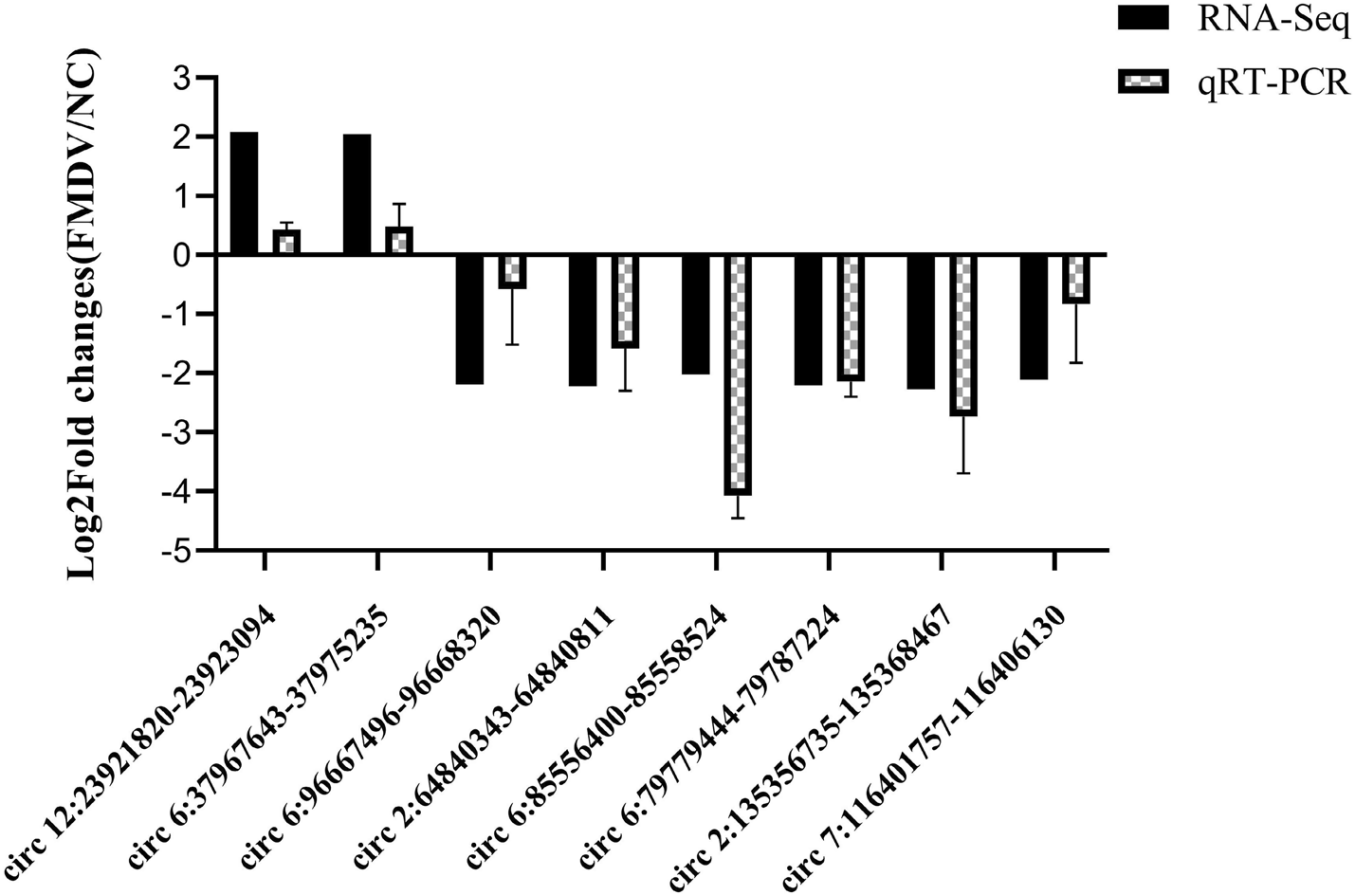
**qRT-PCR validation of differentially expressed circRNAs genes.** PK-15 cells were infected with FMDV for 8 h followed by qRT-PCR to detect differentially expressed circRNAs. Negative control (NC) indicated no infection with FMDV. The *x*-axis indicates the ID of the circRNAs genes, the *y*-axis represents the fold change after FMDV infection, and GAPDH was used as internal control.

To verify the reliability of the RNA-seq data, qRT-PCR was performed to detect circRNAs expression change in PK-15 cells infected with FMDV or not. Eight candidate circRNAs were selected to verify their reliability by qRT-PCR. The primers were designed according to the cirRNAs position (Table 4). The FMDV infected group was compared to uninfected controls, circ12: 23,921,820–23,923,094, circ6: 37,967,643–37,975,235 were up-regulated. The circ6: 96,667,496–96,668,320, circ2:64,840,343–64,840,811, circ7: 116,401,757–116,406,130, circ2: 135,356,735–135,368,467, circ6: 85,556,400–85,558,524, circ6: 79,779,444–79,787,224 were significantly down-regulated (Figure 6). These results indicated that the circRNAs in the RNA-seq datasets were reliable.

**Table 4:** **Primers used in differentially expressed circRNAs validation by qRT-PCR method**

Circ RNA ID	Original gene	Primer type	Divergent primers
12:23921820–23923094	KPNB1	F R	TCCGCCTCCTGGGTTCCCG GTTTTGGGTAGAATGAGGGAGTTTT
6:37967643–37975235	VPS35	F R	TGTACTGATTCCTCATTATGGTTACAA GAGAAAATAGTGTCAAAGATGGGAGGTT
6:96667496–96668320	CEP192	F R	AAAGTAGCCACGCTGCAAGAAATAGAC ATGACAAACAACATTGACCGCAGC
2:64840343–64840811	DDX39A	F R	ACAGAATAAGTGTCCAATGAACTGA GTAAGAGGCACTACCGTGTTTCA
7:116401757–116406130	DICER1	F R	AAATTATTGTTATTTGTAATTATTTATAAC TCCTATTCTTTAGGGCTGATCTCACA
2:135356735–135368467	HSPA4	F R	TTAGTGGCATTAAAAGCAGTGAAAATAG GCTGAGCTAGGAGGATTGCTTGAG
6:85556400–85558524	RCC1	F R	ATTCAGCTTATGAGATAAATCCTGGTT GGTGATAGAGCAAGACTCCATCTCAAAA
6:79779444–79787224	USP48	F R	TTGAGGGCATAAATACCAGGAGGG ACAGCCTCACCCAAACCACCTCAT

## Discussion

As new rising stars of the noncoding RNA field, circRNAs are a fascinating class of RNAs primarily found in the cytoplasm of eukaryotic cells and are considered to be the product of a reverse-splicing reaction during transcription [48]. Circular RNAs have multiple biological functions and are involved in the regulation of gene expression, disease development, and the immune response [49–51]. However, to date, little is known about the characteristics and functions of circRNA expression in host cells during viral infection. With the development of sequencing technology and bioinformatics, scientists have begun to unravel the roles played by circRNAs in host cells following viral infection. It has been shown that host circRNAs change after infection with certain viruses, such as porcine epidemic diarrhea virus (PEDV) [20], avian leukemia virus (ALV) [5], and transmissible gastroenteritis virus (TGEV) [52]. The function of these differentially expressed circRNAs has been analyzed by GO enrichment, KEGG pathways, and ceRNA networks to elucidate their underlying functions and mechanisms during infection.

FMD is a transmissible disease which rapidly spreads over vast areas and causes devastating effects to the livestock industry. In this study, we infected PK-15 cells with FMDV and identified 1100 differentially expressed circRNAs. Through GO and STING analysis, we found that differentially expressed circRNAs were mainly involved in biological processes, such as host cell nucleic acid metabolism and cell cycle regulation induced by FMDV infection, disruption of the cell cycle, affecting the normal growth status of cells, and oncogenesis. Studies have revealed that circPLK1 promotes breast cancer cell proliferation, migration, and invasion in breast cells [53]. Overexpression of circRNACCDC66 promoted the growth and metastasis of colon cancer cells [54]. In this study, differentially expressed circRNAs, circ12: 23921820–23923094 (KPNB1), circ6: 37967643–37975235 (VPS35), circ2: 64840343–64840811 (DDX39A), and circ7: 116401757–116406130 (DICER1) are involved in biological processes, such as DNA damage repair, nucleic acid metabolism, and transport after FMDV infection. The circRNAs circ6: 96667496–96668320 (CEP192), circ6: 85556400–85558524 (RCC1), circRNA7: 39403319–39407636 (CDC5L) are involved in the regulation of the cell cycle. The above results demonstrate that the differentially expressed circRNAs in this study may be involved in the host response to FMDV infection, but the detailed functions and regulatory mechanisms of these circRNAs need to be further explored.

In addition, according to KEGG functional enrichment, most differentially expressed circRNAs were involved in biological processes such as protein ubiquitination degradation, autophagy, mTOR signaling, and cell cycle regulation, which have a close association with the immune response. Ubiquitination is a key signaling mechanism for host cells to deal with viral infections and includes post-translational modifications and proteolytic reactions. The ubiquitin–proteasome pathway is involved in regulating a variety of cellular processes, such as antigen presentation, cell cycle regulation, apoptosis, immune responses, inflammation, and viral infections [55]. Various ubiquitination signals play an important role in the activation of the innate immune response. It has been shown that circRNAs participate in the protein ubiquitination process after viral infection. FMDV was able to disrupt the ubiquitination of proteins through multiple pathways and evade killing by the host immune system. For example, FMDV L^pro^ could target and remove the ubiquitin-like protein, ISG15, and specifically cleave peptide bonds at the C-terminal Gly-Gly sequence to disrupt the ubiquitination modification system and inhibit the initiation of the innate immune response [32]. Orf is a worldwide zoonotic disease caused by Orf virus (ORFV). Following ORFV infection, circRNA regulates the ubiquitination process of host cells and affect the host immune response [56]. In this study, differentially expressed circ6: 79779444–79787224 (USP48) recognized and hydrolyzed the peptide bond at the C-terminal Gly residue of the protein and was involved in the processing of polyubiquitin and ubiquitinated proteins. It was further speculated that circRNA6: 79779444–79787224 is involved in the FMDV L^pro^ mediated immune escape mechanism. Recently, it has been shown that circRNAs are capable of participating in the process of virus-induced autophagy in host cells. The circRNA GATAD2A was able to promote the replication of H1N1 replication by inhibiting host cell autophagy during IAV infection [21]. Overexpression of circNF1-419 regulated cellular autophagy through the PI3K-1/Akt-AMPK-mTOR and PI3K-1/Akt-mTOR signaling pathways in astrocytes, providing a new strategy for the treatment of Alzheimer’s disease [57]. Other studies have shown that viruses can promote their own replication by inhibiting cellular autophagy [58]. FMDV also mediates autophagy to evade innate immunity and promote viral replication through multiple pathways. FMDV inhibits interferon production by inducing PERK-mediated autophagy [30]. FMDV VP2 can also interact with HSPB1 (Heat shock protein family B1) and activate the EIF2S1-ATF4 pathway to inhibit the AKT-MTOR signaling pathway, which inhibits autophagy in host cells to promote viral replication [31]. Heat stress stimulation can induce the synthesis of heat shock proteins which can repair the damaged proteins and degrade the unrepairable proteins as “chaperones,” maintain the stable conformation of proteins, ensure the correct folding of nascent proteins, protect cells against stress damage, enhance the tolerance of cells, and support the normal functional metabolism of cells [59, 60]. In the present study, the circ2: 135356735–135368467 parental gene, HSPA4, belongs to a member of the heat shock protein family, which was significantly downregulated. We hypothesize that circ2: 135356735–135368467 may be involved in FMDV infection-mediated autophagy to evade the host immune response. Therefore, differentially expressed circRNAs in host cells may play an important role in regulating the immune response mediated by viral infection following FMDV infection. The analysis based on the above functions showed that circRNAs play important roles in regulating organism homeostasis during FMDV infection. We further validated the RNA-Seq results by circRNAs qRT-PCR. The obtained data showed a similar expression pattern compared with RNA-seq. These results indicated that the differential expression of circRNAs seen in the RNA-seq datasets was reliable.

CircRNAs are usually generated by back-splicing of pre-mRNA and exhibit strong structural stability and spatiotemporal specificity [2, 3, 61]. Interestingly, we found that a single gene locus could be used to generate one or more circRNAs through alternative splicing. Our results show that multiple circRNA subtypes derived from the same genes were differentially expressed after FMDV infection in PK-15 cells. These circRNA parental genes play an important role in regulating the spatiotemporal expression of circRNAs.

The regulation of miRNA target gene expression by circRNAs as miRNA sponges is a classical mechanism of the ceRNAs hypothesis [62, 63]. Most circRNAs act as miRNAs sponges to regulate gene expression [7, 17]. ciRS-7 functions as a miR-7 sponge and contains more than 70 miRNA binding targets that inhibit miR-7 activity and promote miR-7 target gene expression [64]. The development of metastases in pancreatic cancer was associated with ciRS-7 regulating miR-7-mediated EGFR/STAT3 signaling [65]. During ORFV infection, circRNAs act as miRNA sponges to generate circRNA-miRNA-mRNA networks that indirectly regulate gene expression following ORFV infection [56]. CircRNAs have also been shown to have essential regulatory roles in colon cancer [54], breast cancer [66], and liver cancer [67]. In addition, because of the unique mode of action of circRNAs, which are stable and enriched in cells, they may be useful as molecular markers for the diagnosis of ALV and HBV infections [5, 68].

In this study, we identified and analyzed the functions of differentially expressed circRNAs in host cells after FMDV infection based on GO and KEGG functional enrichment analysis, but we did not predict the miRNA target sites and miRNA target genes of the differentially expressed circRNAs. The detailed functions and regulatory mechanisms of these differentially expressed circRNAs in viral infection or the cell cycle will be the subject of subsequent studies.

In summary, we found that FMDV infection resulted in the differential expression of circRNAs within host cells based on GO and KEGG functional enrichment analysis. The results suggest that these circRNAs are involved in the regulation of host immune response processes. In addition, this study was the first to analyze expression profiles of differential circRNAs and the biological function of parental genes derived from FMDV-infected host cells. This provides new insights for researchers to understand the mechanism underlying FMDV–host interactions from the perspective of circRNAs.
